# A Narrative Review of Patient-Reported Outcome Measures and Their Application in Recent Pediatric Surgical Research: Advancing Knowledge and Offering New Perspectives to the Field

**DOI:** 10.1055/s-0043-1778108

**Published:** 2024-01-25

**Authors:** Olivia K.C. Spivack, Michaela Dellenmark-Blom, Jens Dingemann, Chantal A. ten Kate, Vuokko Wallace, Wichor M. Bramer, Julia H. Quitmann, Andre Rietman

**Affiliations:** 1 Department of Pediatric Surgery, Erasmus MC Sophia Children's Hospital, Rotterdam, the Netherlands; 2 Quality of Life working group, European Reference Network for rare Inherited Congenital Anomalies (ERNICA); 3 Department of Pediatrics, University of Gothenburg Institute of Clinical Sciences, Gothenburg, Sweden; 4 Department of Pediatric Surgery, Sahlgrenska University Hospital Queen Silvia Children's Hospital, Gothenburg, Sweden; 5 Department of Women's and Children's Health, Karolinska Institute, Stockholm, Sweden; 6 Department of Pediatric Surgery, Hannover Medical School, Hannover, Niedersachsen, Germany; 7 EAT (Esophageal Atresia Global Support Groups), Stuttgart, Germany; 8 Department of Psychology, University of Bath, Bath, United Kingdom; 9 Medical Library, Erasmus Medical Center, Rotterdam, the Netherlands; 10 Faculty of Business and Social Sciences, Hamburg University of Applied Sciences (HAW Hamburg), Hamburg, Germany; 11 Department of Child and Adolescent Psychiatry/Psychology, Erasmus MC Sophia Children's Hospital, Rotterdam, the Netherlands

**Keywords:** patient outcomes, children, congenital anomalies, health status, quality of life

## Abstract

**Introduction** Patient-reported outcome measures (PROMs) can be employed in both research and clinical care to enhance our understanding of outcomes that matter to patients. This narrative review aims to describe PROM use in recent pediatric surgical research, identify and describe psychometrically robust PROMs, providing an overview of those derived from pediatric patient input, and make recommendations for future research.

**Materials and Methods** A search was conducted to identify articles published from 2021 to August 2023 describing the availability and/or use of at least one valid or reliable PROM in children with conditions including anorectal malformations, biliary atresia, congenital diaphragmatic hernia, duodenal atresia, esophageal atresia, abdominal wall defects, Hirschsprung's disease, sacrococcygeal teratoma, and short bowel syndrome. Articles were categorized based on their objectives in applying PROMs. Psychometrically robust PROMs were identified and described.

**Results** Out of the 345 articles identified, 49 met the inclusion criteria. Seventeen focused on esophageal atresia and 14 on Hirschsprung's disease. Twenty-nine PROMs were identified, with 12 deemed psychometrically robust. Seven psychometrically robust PROMs were developed using patient input in the primary item generation. Most PROMs were applied to advance understanding of conditions and/or treatment and fewer were developed or psychometrically evaluated. No PROMs were assessed for their impact or incorporated into an implementation study.

**Conclusions** This review reveals gaps in the application of PROMs in recent pediatric surgical research. Emphasis should be placed on the development and utilization of psychometrically robust PROMs, broadening the scope of covered diseases, conducting impact assessments, and evaluating implementation strategies.

## Introduction

Pediatric surgeons treat children with a variety of low-prevalence and complex congenital conditions. These conditions encompass anomalies involving the esophagus and/or respiratory organs (congenital diaphragmatic hernia, esophageal atresia), bile ducts (as seen in biliary atresia), and the abdomen and bowel/intestine (encompassing anorectal malformations, duodenal atresia, gastroschisis, omphalocele, Hirschsprung's disease, and short bowel syndrome). 1 Children born with these congenital conditions often require surgery to ensure their survival and need inpatient post-surgical care.

Over the past few decades, medical and surgical advances have led to an increased survival rate for children with these conditions. This progress has given rise to a new generation of survivors who reach childhood milestones, adolescence, and adulthood. 2 3 4 5 However, as mortality rates have declined, there is an increasing recognition of the long-term somatic and psychosocial challenges. These challenges encompass both condition-specific morbidities and the impact of long-term medical care, which may involve numerous and sometimes long-term hospitalizations, repeated invasive medical assessments, and anesthesia. 6 Consequently, the focus of outcome assessment in pediatric surgery has shifted from merely assessing mortality to considering the long-term morbidity and patient-reported outcomes using patient-reported outcome measures (PROMs). 1 7 8 9 10 11

In the field of pediatric surgery, the utilization of PROMs, 11 including those measuring multidimensional concepts like health-related quality of life (HRQoL), 1 7 8 9 10 has seen an increase in recent years. In their recent systematic review, Besner et al 11 provide an overview of frequently used, valid PROMs employed to assess components of HRQoL in pediatric surgical research from 1996 to May 2021. In addition to validity, a key psychometric property that can be used to judge the quality of a PROM and the information it collects is reliability. 12 Incorporating direct patient input into the primary item generation phase of PROM development is also recommended as a current standard, 13 to ensure the instrument is comprehensive and measures aspects of importance to the target population. As the field of PROMs in pediatric surgery is growing, it is also important to review PROM development and validation studies, which has not yet been done.

This narrative review therefore aims to further advance our knowledge of available PROMs in pediatric surgery, by describing recent studies in the field, identifying and describing psychometrically robust (valid and reliable) PROMs, and providing an overview of those derived from pediatric patient input. In doing so, this review offers new perspectives and essential recommendations to advance PROM research in pediatric surgery and facilitate successful and effective utilization.

## Methods

### Definitions and Purpose

A patient-reported outcome is information about a patient's health directly provided by the patient, including details about symptoms, health status, quality of life, and the impact of disease and treatment on physical, social, and psychological well-being, known as HRQoL. 13 The tool used to gather this information from patients is called a PROM. 13

PROMs can be categorized as generic or condition-specific, depending on whether they are designed for broader use or tailored to a specific population and clinical context. They can also focus on specific symptoms or domains, allowing for sensitive measurements tailored to different patient groups. 14 15 16 17 In cases where pediatric patients are unable to provide their own reports, caregivers can complete PROMs on their behalf, known as proxy-reports, which provide valuable information about a child's health. 14 18 PROMs help to ensure that care, research, and policymaking remain patient-centered. 15 19 To be considered suitable for use, PROMs must exhibit psychometric robustness, including validity and reliability. 12 In clinical practice, there is growing evidence that the use of pediatric PROMs positively impacts patient outcomes, health processes, and health services, 20 21 22 which is particularly valuable in pediatric surgery due to the risk of long-term and complex morbidity. Implementing PROMs in clinical practice necessitates overcoming methodological challenges, 23 ethical considerations, 24 and potential barriers. 22 24 Effective strategies exist to overcome potential barriers. 22

### Study Design and Search Strategy

We conducted a narrative review to identify studies describing the availability and use of PROMs for children undergoing pediatric surgery. This review adhered to the six predefined criteria aimed at enhancing the quality of narrative reviews, as outlined by Baethge et al. 25 This includes providing an explanation of the article's importance, defining and describing the aims of the review and the literature search, referencing, and presenting appropriate evidence and relevant endpoint data.

A comprehensive search was conducted in the following databases with the assistance of a medical librarian (W.M.B.): Medline (Ovid), CINAHL (EBSCOHost), Embase (embase.com), Web of Science Core Collection, and the Cochrane Central register of Controlled Trials (Wiley). The search strings used in each of the databases are included in Supplementary Material S1 . The search terms were based on previous studies 11 20 and input from four authors in the field of pediatric surgery (J.D., M.D.B., O.K.C.S., A.R.). The initial search had no restrictions on publication dates. However, to extend and advance the systematic review by Besner et al 11 with the latest literature, the identified articles were subsequently filtered to include only those published from January 2021 to August 8, 2023.

### Article Selection

Table 1 presents the inclusion and exclusion criteria for article selection. To identify articles that qualified for inclusion, one researcher (O.K.C.S.) screened the titles, abstracts, and full texts (as necessary) of the publications yielded by the initial search and screened the full texts of all eligible articles. Any uncertainties regarding inclusion or exclusion were discussed with two additional researchers (M.D.B., J.D.).

**Table 1:** Inclusion and exclusion criteria

*Inclusion criteria*
Full-text publication in English language
Published from January 2021 to August 8, 2023
Study design: empirical research using quantitative or qualitative design
Children's data (≤ 18 years) analyzed separately from those of the adult population when both children and adults were assessed
Patient-reported outcome measurement (PROM) ● Used to advance knowledge of the condition and/or treatment ● Development and psychometric evaluation ● Assessment of PROM impact ● Use in implementation studies (e.g., attempts to understand factors influencing implementation outcomes, use of implementation strategies, and process and outcome evaluation)
Conditions treated in pediatric surgery: ● Anorectal malformations ● Biliary atresia ● Congenital diaphragmatic hernia ● Duodenal atresia ● Esophageal atresia/tracheoesophageal fistula ● Abdominal wall defects: gastroschisis; omphalocele ● Hirschsprung's disease ● Sacrococcygeal teratoma ● Short bowel syndrome
*Exclusion criteria*
Participant group solely composed of adults (over 18 years of age)
Studies only using self-developed patient-reported outcome measurement without psychometric evaluation for validity or reliability
Studies only using PROMs originally developed for adults and used in children without psychometric evaluation for validity or reliability
Studies only using PROMs developed for other conditions than the study population in which it was applied, without psychometric evaluation for validity or reliability
Questionnaires assessing cognitive ability and intelligence
Literature reviews
Not succeeded in retrieving full-text publication

Abbreviation: PROMs, patient-reported outcome measures.

Articles were included if they employed at least one valid or reliable PROM 26 and assessed aspects of physical, mental, social, or multidimensional concepts, including HRQoL, in children aged 18 years or younger with conditions including anorectal malformations, biliary atresia, congenital diaphragmatic hernia, duodenal atresia, esophageal atresia, abdominal wall defects (omphalocele, gastroschisis), Hirschsprung's disease, sacrococcygeal teratoma, and short bowel syndrome. The PROM results needed to be sufficiently analyzed and clearly reported for at least one of these patient populations. Studies assessing patients' health status using single yes/no response questions were not regarded as employing a valid or reliable PROM and were therefore excluded.

For a PROM to be deemed “valid,” a validation process needed to have been carried out in a pediatric population either by (1) evaluating associations between children's scores on the instrument and independent measures of similar constructs, (2) comparing scores among groups of children expected to differ on patient-reported outcomes (e.g., healthy children compared to those with chronic conditions), or (3) evaluating the instrument to assess the construct (structural) validity of its scale(s). PROMs were considered reliable if they demonstrated a good inter-item relationship between the items (internal consistency), stability of scores (retest reliability), and/or interrater reliability. 26

### Data Extraction and Data Analysis

The following information from the articles was extracted by one author (O.K.C.S.) and subsequently reviewed by two additional authors (M.D.B., A.R.). The extracted data included the year of publication, the country or countries of origin, the study design (observational/experimental), the specific condition(s) under investigation, the number and type of PROMs employed, and the intended application of the PROMs. When determining the number of instruments, variations such as age-specific or self/parent-proxy versions, as well as short and long versions of the same instrument, were counted as a single instrument. Articles and their included PROMs were categorized into one of five groups based on their intended application. These encompassed the following:

- Studies primarily focused on developing a PROM.
- Studies conducting a psychometric evaluation of an existing PROM.
- Studies utilizing PROMs to advance knowledge of the condition and/or treatment being investigated.
- Studies assessing the impact of PROM utilization on patient, process, and/or health service-related outcomes in clinical practice.
- Implementation studies, which included studies aimed at understanding the determinants influencing PROM implementation, studies applying strategies to promote successful and effective implementation in clinical practice, and studies conducting process and outcome evaluations following implementation efforts.

### Assessment of Psychometric Robustness

**Fig. 1:**
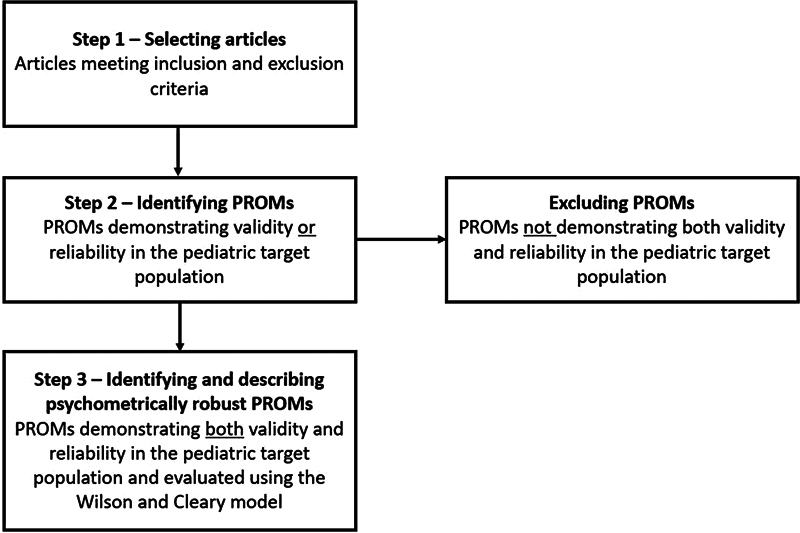
PROM identification process.

To identify psychometrically robust PROMs for use in pediatric surgery, one author (M.D.B.) either accessed referenced articles linked to the chosen PROM, consulted the Medline database, or consulted the PROM's webpage. PROMs were considered psychometrically robust if they demonstrated both validity and reliability in the pediatric target population, in accordance with the definitions previously outlined. The findings were discussed with two authors (J.D., A.R.) to reach consensus. The psychometrically robust PROMs were categorized by one author (M.D.B.) into the following PROM types and reviewed by a second author (A.R.): generic, condition-specific, symptom-specific, or domain-specific (e.g., assessing a specific area of HRQoL or function). The authors M.D.B. and A.R. jointly evaluated whether a PROM's items were derived from initial patient input (yes/no) 26 and applied the Wilson and Cleary model 27 to classify the primary conceptual content of all PROMs deemed psychometrically robust. This model assesses whether a PROM encompasses aspects related to biological and physiological factors, symptom status, functioning, general health perceptions, and overall quality of life. Additionally, the number of articles in which such psychometrically robust PROMs were applied was determined. See Fig. 1 for an overview of the PROM identification process.

## Results

Out of 345 articles identified by the database searches, 49 articles met the inclusion criteria. 28 29 30 31 32 33 34 35 36 37 38 39 40 41 42 43 44 45 46 47 48 49 50 51 52 53 54 55 56 57 58 59 60 61 62 63 64 65 66 67 68 69 70 71 72 73 74 75 76 Most concerned esophageal atresia ( *n* = 17) and Hirschsprung's disease ( *n* = 14). None of the articles focused on duodenal atresia or omphalocele. PROM studies were conducted in Europe ( *n* = 32), Asia ( *n* = 8), North America (United States, *n* = 7), and Australia ( *n* = 2), but not in South America or Africa ( *n* = 0). Three studies were multi-country studies. Comprehensive information is provided in Table 2 .

**Table 2:** Selected articles categorized per condition studied and publication year 28 29 30 31 32 33 34 35 36 37 38 39 40 41 42 43 44 45 46 47 48 49 50 51 52 53 54 55 56 57 58 59 60 61 62 63 64 65 66 67 68 69 70 71 72 73 74 75 76

Author and year	Country/countries	Study design	Patient-reported outcome measure(s) used (as reported in the article)	Aim and intended application of the patient-reported outcome measure(s) used
Anorectal malformations				
Arneitz et al, 2021 28	Austria	Observational	Rintala Bowel Function Score Quality-of-Life Scoring Criteria for Children with Fecal Incontinence	To assess quality of life and fecal continence in relation to the individual sports performance in anorectal malformation patients *Intended application* : to advance knowledge of a condition and/or treatment
Wood et al, 2021 29	United States of America (United States)	Observational	Baylor Continence Scale questionnaire Vancouver Symptom Score questionnaire (urinary symptoms) Cleveland Constipation Scoring System questionnaire Pediatric Quality-of-Life Inventory 4.0 generic core scales	To objectively assess the effects of a bowel management program *Intended application* : to advance knowledge of a condition and/or treatment
Sharifi-Rad et al, 2022 30	Iran	Observational	Baylor Continence Scale questionnaire Fecal Incontinence Quality of Life questionnaire	To assess the effectiveness of a pelvic floor rehabilitation program including transcutaneous functional electrical stimulation (TFES) in combination with pelvic floor muscle exercises *Intended application* : to advance knowledge of a condition and/or treatment
Tofft et al, 2022 31	Sweden	Observational	Pediatric Quality-of-Life Inventory 4.0 generic core scales	To assess the psychosocial significance of abdominal scarring in anorectal malformations *Intended application* : to advance knowledge of a condition and/or treatment
Biliary atresia				
Liang et al, 2021 32	China	Observational	Pediatric Quality-of-Life Inventory 4.0 generic core scales In-house questionnaire (post-surgical status)	To investigate the post-surgical status and status of children's quality of life after biliary atresia treatment *Intended application* : to advance knowledge of a condition and/or treatment
Congenital diaphragmatic hernia				
Tragesser et al, 2021 33	United States	Observational	Pediatric Quality-of-Life Inventory 4.0 generic core scales In house survey regarding various aspects of the child's care, including functional status	To characterize the barriers to follow-up care that congenital diaphragmatic hernia patients face *Intended application* : to advance knowledge of a condition and/or treatment
Azab et al, 2023 34	Saudi Arabia	Experimental	Pediatric Quality-of-Life Inventory 4.0 generic core scales	To explore the effects of virtual reality-based exercise programs on quality of life in children with repaired congenital diaphragmatic hernia *Intended application* : to advance knowledge of a condition and/or treatment
Sreeram et al, 2023 35	The Netherlands	Observational	Pediatric Quality-of-Life Inventory 4.0 generic core scales Dutch-Child-AZL-TNO-Quality-of-Life questionnaire	To longitudinally evaluate self-reported health status and quality of life in 8- and 12-year-old survivors of congenital diaphragmatic hernia *Intended application* : to advance knowledge of a condition and/or treatment
Esophageal atresia				
Gallo et al, 2021 36	The Netherlands	Observational	Gastrointestinal Quality of Life Index Child Health Questionnaire (CHF87-BREF) TNO-AZL Questionnaire for Children's Health-Related Quality of Life	To assess the quality of life after esophageal replacement for long gap esophageal atresia patients *Intended application* : to advance knowledge of a condition and/or treatment
Soyer et al, 2021 37	Turkey	Observational	Esophageal-Atresia-Quality-of-life Questionnaires Pediatric Quality-of-Life Inventory 4.0 generic core scales	To report the feasibility, validity, and reliability of the Turkish versions of the Esophageal-Atresia-Quality-of-Life (EA-QOL) questionnaires, which were originally developed in Sweden and Germany *Intended application* : psychometric evaluation of an existing PROM
Tan Tanny et al, 2021 38	Australia	Observational	Pediatric Quality-of-Life Inventory 4.0 generic core scales	To investigate the quality-of-life impact on patients *Intended application* : to advance knowledge of a condition and/or treatment
Ten Kate et al, 2021 39	The Netherlands	Observational	Pediatric Quality-of-Life Inventory 4.0 generic core scales Dutch-Child-AZL-TNO-Quality-of-Life questionnaire	To evaluate self-reported and proxy-reported health status and quality of life of school-aged children born with esophageal atresia *Intended application* : to advance knowledge of a condition and/or treatment
van Tuyll van Serooskerken et al, 2021 40	The Netherlands	Observational	Gastroesophageal Reflux Symptom Questionnaire Reflux Disease Questionnaire Pediatric Quality-of-Life Inventory 4.0 generic core scales	To evaluate the childhood outcome in long gap esophageal atresia patients treated with the thoracoscopic external traction technique, including gastrointestinal outcome and HRQoL *Intended application* : to advance knowledge of a condition and/or treatment
Witt et al, 2021 41	Germany	Observational	Strengths and Difficulties Questionnaire	To measure internalizing and behavioral problems of children and adolescents with esophageal atresia *Intended application* : to advance knowledge of a condition and/or treatment
Witt et al, 2021 42	Germany and Sweden	Observational	Pediatric Quality-of-Life Inventory 4.0 generic core scales Esophageal-Atresia-Quality-of-life Questionnaires	To compare parent and child-reported HRQoL of children born with esophageal atresia *Intended application* : to advance knowledge of a condition and/or treatment
Dellenmark-Blom et al, 2022 43	Sweden	Observational	Strengths and Difficulties Questionnaire Pediatric Quality-of-Life Inventory 4.0 generic core scales Esophageal-Atresia-Quality-of-life Questionnaires	To identify the prevalence of mental health problems in children with long-gap esophageal atresia and HRQoL *Intended application* : to advance knowledge of a condition and/or treatment
Dellenmark Blom et al, 2022 44	Sweden	Observational	Pediatric Quality-of-Life Inventory 4.0 generic core scales Esophageal-Atresia-Quality-of-life Questionnaires	To evaluate HRQoL in a Swedish national cohort of children with delayed reconstruction of esophageal atresia *Intended application* : to advance knowledge of a condition and/or treatment
Dellenmark Blom et al, 2022 45	Sweden and Germany	Observational	Pediatric Quality-of-Life Inventory 4.0 generic core scales	To identify factors (including the child's quality of life) related to family impact in children with esophageal atresia *Intended application* : to advance knowledge of a condition and/or treatment
Di Natale et al, 2022 46	Switzerland	Observational	KIDSCREEN-27	To assess HRQoL among young patients after esophageal atresia repair *Intended application* : to advance knowledge of a condition and/or treatment
Li et al, 2022 47	China	Observational	Esophageal-Atresia-Quality-of-Life Questionnaires	To evaluate the linguistic and content validity of the Chinese Mandarin version of the Esophageal Atresia Quality of Life (EA-QOL) questionnaires, which were originally developed in Sweden and Germany *Intended application* : psychometric evaluation of an existing PROM
Mikkelsen et al, 2022 48	Norway	Observational	Pediatric Quality-of-Life Inventory 4.0 generic core scales Strengths and Difficulties Questionnaire	To investigate quality of life in esophageal atresia patients in relation to comparison groups and to clinical factors including mental health *Intended application* : to advance knowledge of a condition and/or treatment
Rozensztrauch et al, 2022 49	Poland	Observational	Esophageal-Atresia-Quality-of-Life Questionnaires	To evaluate the reliability and validity of the Polish version of the Esophageal Atresia Quality of Life (EA-QOL) questionnaires, which were originally developed in Sweden and Germany *Intended application* : psychometric evaluation of an existing PROM
Ten Kate et al, 2022 50	The Netherlands	Observational	Esophageal-Atresia-Quality-of-Life Questionnaires	To evaluate the psychometric performance of the Esophageal Atresia Quality of Life (EA-QOL) questionnaires in Dutch children, which were originally developed in Sweden and Germany *Intended application* : psychometric evaluation of an existing PROM
Boettcher et al, 2023 51	Germany	Observational	Pediatric Quality-of-Life Inventory 4.0 generic core scales, short form-15 Strengths and Difficulties Questionnaire	To evaluate quality of life and mental health of patients with esophageal atresia *Intended application* : to advance knowledge of a condition and/or treatment
Örnö Ax et al, 2023 52	Sweden	Observational	Pediatric Quality-of-Life Inventory 4.0 generic core scales	To investigate the association of feeding difficulties and generic HRQoL among children aged 2–7 and 8–17 years, born with esophageal atresia *Intended application* : to advance knowledge of a condition and/or treatment
Gastroschisis				
De Bie et al, 2021 53	United States	Observational	Pediatric Quality-of-Life Inventory 4.0 generic core scales Pediatric Quality-of-Life Inventory Cognitive Functioning Scale Pediatric Quality-of-Life Inventory Gastrointestinal Symptoms Scale Survey capturing a parental subjective evaluation of their child's overall quality of life	To evaluate the long-term outcomes of a homogenous patient population, reporting the standardized core outcome set developed specifically for gastroschisis (including quality of life) *Intended application* : to advance knowledge of a condition and/or treatment
Hirschsprung's disease				
Byström et al, 2021 54	Sweden	Observational	Rintala Bowel Function Score Lower Urinary Tract symptoms questionnaire KIDSCREEN-52	To evaluate long-term bowel function, lower urinary tract symptoms, and quality of life in patients treated for Hirschsprung's disease with transanal endorectal pull-though (TERPT) compared with healthy controls *Intended application* : to advance knowledge of a condition and/or treatment
Davidson et al, 2021 55	United Kingdom	Observational	Rintala Bowel Function Score Pediatric Quality-of-Life Inventory 4.0 generic core scales Modified Danish Prostatic Symptom Score	To describe functional and HRQoL outcomes in patients with Hirschsprung's disease with associated learning disability or neurodevelopmental delay, completing a core outcome set for Hirschsprung's disease *Intended application* : to advance knowledge of a condition and/or treatment
Davidson et al, 2021 56	United Kingdom	Observational	Pediatric Quality-of-Life Inventory 4.0 generic core scales Rintala Bowel Function Score 8-item questionnaire adapted from the Danish Prostatic Symptom Score (lower urinary tract symptoms)	To describe detailed long-term operative and patient-reported outcomes for bowel and urologic function and HRQoL with comparison to previously published normative data, including all domains of the recently developed core outcome set for Hirschsprung's disease *Intended application* : to advance knowledge of a condition and/or treatment
Loganathan et al, 2021 57	India	Observational	Pediatric Quality-of-Life Inventory 4.0 generic core scales Rintala Bowel Function Score	To assess the general and condition-specific quality of life in children treated for Hirschsprung's disease from a developing country *Intended application* : to advance knowledge of a condition and/or treatment
Liu et al, 2021 58	China	Observational	Pediatric Quality-of-Life Inventory 4.0 generic core scales Wexner Scoring System (Defecation function)	To explore the application effect (e.g., on quality of life and defecation function) of trinity new model home nursing in postoperative management of children with Hirschsprung's disease *Intended application* : to advance knowledge of a condition and/or treatment
Davidson et al, 2022 59	Finland and United Kingdom	Observational	Rintala Bowel Function Score Modified Danish Prostatic Symptom Score (urinary symptoms) Pediatric Quality-of-Life Inventory 4.0 generic core scales	To compare Duhamel and endorectal pull-through with a cross-sectional assessment of outcomes in relation to matched normal population controls in contemporaneous, age-matched cohorts of patients with Hirschsprung's disease at two large-volume referral centers *Intended application* : to advance knowledge of a condition and/or treatment
Diez et al, 2022 60	Germany	Experimental	KINDL _R_ (KINDL-R)	To compare the clinical efficacy (including quality of life) of noninvasive sacral neuromodulation and conventional therapeutic options *Intended application* : to advance knowledge of a condition and/or treatment
Miyano et al, 2022 61	Japan	Observational	Child Health Questionnaire Child Form 87 Rintala Bowel Function score Quality-of-Life Score (QoLS) modified for use	To assess mid-/long-term quality of life of total colonic aganglionosis patients by assessing general lifestyle, bowel function, and mental health from childhood to adulthood with respect to pull through technique and compare subject responses with their caregivers' responses *Intended application* : to advance knowledge of a condition and/or treatment
Tham et al, 2022 62	United States	Observational	Pediatric Quality-of-Life Inventory 4.0 generic core scales Baylor Social Continence Scale	To compare parent-proxy versus child self-report HRQoL in children with Hirschsprung's disease to children with functional constipation (FC) and examine predictors of HRQoL *Intended application* : to advance knowledge of a condition and/or treatment
Verkuijl et al, 2022 63	The Netherlands	Observational	Early Pediatric Groningen Defecation and Fecal Continence questionnaire Child Health Questionnaire Child Form 87	To compare long-term bowel function and generic quality of life between patients with familial and nonfamilial Hirschsprung's disease *Intended application* : to advance knowledge of a condition and/or treatment
Verkuijl et al, 2022 64	The Netherlands	Observational	Early Pediatric Groningen Defecation and Fecal Continence questionnaire Child Health Questionnaire Child Form 87	To compare long-term bowel function and generic quality of life in Hirschsprung's disease patients with total colonic or long-segment versus rectosigmoid aganglionosis *Intended application* : to advance knowledge of a condition and/or treatment
Koo et al, 2023 65	Australia	Observational	Pediatric Quality-of-Life Inventory 4.0 generic core scales	(1) Determine whether the HRQoL of children with Hirschsprung's disease differs from healthy pediatric populations and (2) explore the relationship between children's HRQoL and psychosocial outcomes of parents *Intended application* : to advance knowledge of a condition and/or treatment
Telborn et al, 2023 66	Sweden	Observational	Diet and Bowel Function Questionnaire Rintala Bowel Function Score	To develop a patient-reported outcome instrument, for children with and without Hirschsprung's disease, to explore experiences of dietary effects on bowel function *Intended application* : development of a PROM
Zhang et al, 2023 67	China	Observational	Pediatric Quality-of-Life Inventory 4.0 generic core scales Rintala Bowel Function Score	To define controlled outcomes for bowel function and quality of life after transanal rectal mucosectomy and partial internal anal sphincterotomy pull-through (TRM-PIAS, a modified Swenson procedure) for Hirschsprung's disease. *Intended application* : to advance knowledge of a condition and/or treatment
Sacrococcygeal teratoma				
Mehl et al, 2022 68	United States	Observational	Pediatric Quality-of-Life Inventory 4.0 generic core scales Baylor Continence Scale	To evaluate the relationship of social continence with patient centered outcomes, such as quality of life, in children with sacrococcygeal teratoma *Intended application* : to advance knowledge of a condition and/or treatment
Short bowel syndrome				
Silva et al, 2022 69	Portugal	Observational	Pediatric Quality-of-Life Inventory 4.0 generic core scales	To assess impact of short bowel syndrome on quality of life for children *Intended application* : to advance knowledge of a condition and/or treatment
Multiple conditions				
Darmaun et al, 2021 70	France	Observational	Pediatric Quality-of-Life Inventory 4.0 generic core scales	To assess quality of life in children with congenital diaphragmatic hernia and to compare it with esophageal atresia *Intended application* : to advance knowledge of a condition and/or treatment
Fuerboeter et al, 2021 71	Germany	Observational	Pediatric Quality-of-Life Inventory 4.0 generic core scales Strengths and Difficulties Questionnaire	To evaluate the HRQoL and mental health of children with rare congenital surgical diseases, including anorectal malformations, biliary atresia, congenital diaphragmatic hernia, esophageal atresia, and Hirschsprung's disease *Intended application* : to advance knowledge of a condition and/or treatment
Judd-Glossy et al, 2021 72	United States	Observational	Strengths and Difficulties Questionnaire	To evaluate the psychosocial functioning of patients with anorectal malformation and Hirschsprung's disease during the beginning of participation in a bowel management program *Intended application* : to advance knowledge of a condition and/or treatment
Stathopoulos et al, 2021 73	United Kingdom	Observational	Gastrointestinal functional outcomes assessment using Krickenbeck scoring Modified Hirschsprung's disease anorectal malformation quality of life questionnaire	To assess bowel function and quality of life in children and adolescents with congenital colorectal malformations (anorectal malformation, Hirschsprung's disease) during the first UK COVID lockdown period *Intended application* : to advance knowledge of a condition and/or treatment
Sreeram et al, 2021 74	The Netherlands	Observational	Pediatric Quality-of-Life Inventory 4.0 generic core scales Dutch-Child-AZL-TNO-Quality-of-Life questionnaire	To assess the added value of self-reported PROMs for health status and quality of life in the long-term follow-up of children with foregut anomalies, including congenital diaphragmatic hernia and esophageal atresia *Intended application* : to advance knowledge of a condition and/or treatment
Ilik et al, 2022 75	The Netherlands	Observational	Pediatric Perceived Cognitive Functioning Questionnaire	To evaluate the additional value of the Pediatric Perceived Cognitive Functioning Questionnaire (PedsPCF) within a follow-up program for children with congenital foregut anatomical anomalies and/or neonatal ECMO treatment by assessing the association between the PedsPCF and the frequently used behavior rating inventory of executive function and the association between the PedsPCF and neuropsychological assessments *Intended application* : to advance knowledge of a condition and/or treatment
Baaleman et al, 2023 76	United States	Observational	Pediatric Quality-of-Life Inventory Gastrointestinal Symptoms Module	To investigate long-term outcomes of antegrade continence enema (ACE) treatment (including on quality of life) in children with anorectal malformations and Hirschsprung's disease *Intended application* : to advance knowledge of a condition and/or treatment

Abbreviations: HRQoL, health-related quality of life; PROM, patient-reported outcome measure.

The 49 articles identified a total of 29 PROMs. 66 77 78 79 80 81 82 83 84 85 86 87 88 89 90 91 92 93 94 95 96 97 98 99 100 101 102 103 104 105 The most frequently used PROM, applied predominantly in patients with esophageal atresia ( *n* = 11) or Hirschsprung's disease ( *n* = 8), was the Pediatric Quality-of-Life Inventory 4.0 generic core scales (PedsQL 4.0) 83 ( *n* = 31). Four common assessments for colorectal function, including (in)continence, in anorectal malformations and/or Hirschsprung's disease were the Rintala Bowel Function Scoring System 77 ( *n* = 9), Baylor Continence Scale (BCS) 78 ( *n* = 4), Cleveland Clinic Fecal Incontinence Severity Scoring System (or Wexner score) 81 82 ( *n* = 2), and the Krickenbeck criteria 102 ( *n* = 1).

**Fig. 2:**
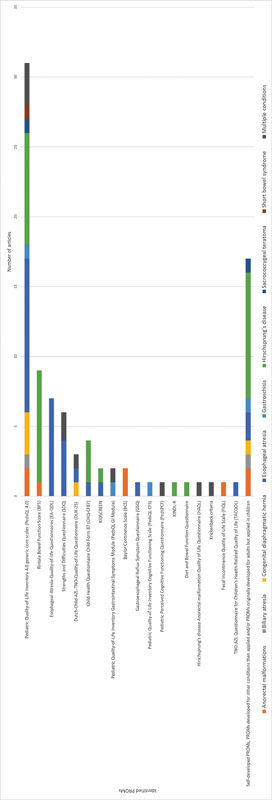
The number of articles employing the identified PROMs, categorized per condition.

Seventeen studies employed PROMs that were self-developed, developed for other conditions than applied and/or originally developed for adults. Five of these studies employed PROMs that were self-developed. Four studies employed PROMs that were developed for other conditions than applied, including the Vancouver Symptom Score, 79 the BCS, 78 when applied in Hirschsprung's disease and sacrococcygeal teratoma patient populations, and the Lower Urinary Tract Symptoms (LUTS) Questionnaire 80 (see Table 2 ). Nine studies employed PROMs originally developed for adults ( *n* = 5), including the Gastrointestinal Quality of Life Index (GIQLI), 92 Cleveland Clinic Fecal Incontinence Severity Scoring System (or Wexner score), 81 82 Reflux Disease Questionnaire (RDQ), 93 Danish Prostatic Symptom Score (DAN-PSS), 99 and the Early Pediatric Groningen Defecation and Fecal Continence Questionnaire (EP-DeFeC). 101 Mental health assessments were conducted using the Strengths and Difficulties Questionnaire (SDQ) 94 in studies focusing on esophageal atresia ( *n* = 4) and on more than one condition ( *n* = 2). Fig. 2 presents the number of articles employing the identified PROMs, categorized per condition.

Out of the 29 PROMs identified, most ( *n* = 28) were applied in observational studies ( *n* = 47), while only two studies employed an experimental, randomized trial design. 34 60 The majority of articles employed PROMs to enhance understanding of the respective medical conditions and treatment strategies under investigation ( *n* = 44). One study was dedicated to the development of a specific PROM, known as the Diet and Bowel Questionnaire. 66 Four studies focused on the psychometric evaluation of one condition-specific PROM (Esophageal-Atresia-Quality-of-life Questionnaires, EA-QOL) across four countries. 37 47 49 50 None of the PROMs were subjected to an evaluation of their impact on patient, process, and/or health service-related outcomes. However, three PROMs were applied, in two studies, 74 75 to assess the relationship between the PROM of interest and other outcome measures, aiming to determine its added value in follow-up care. No PROM was applied as part of an implementation study.

Table 3 lists the 12 PROMs that were deemed psychometrically robust. These PROMs were found to demonstrate both validity and reliability in their pediatric target population, while remaining aligned with the instrument's original conceptual aim. Six of these psychometrically sound PROMs were generic HRQoL questionnaires, 83 85 87 88 89 90 95 100 two were condition-specific HRQoL questionnaires for children born with esophageal atresia (EA-QOL) 91 or Hirschsprung's disease and anorectal malformations (Hirschsprung's disease Anorectal malformation Quality of Life Questionnaire, HAQL), 103 and four were symptom-specific or domain-specific, targeting mental health (SDQ), 94 gastrointestinal symptoms (PedsQL Gastrointestinal Symptoms [GI] Module) 98 or cognitive functioning (PedsQL Cognitive Functioning Scale [PedsCFS] 96 97 and Pediatric Perceived Cognitive Functioning Questionnaire [PedsPCF] 104 105 ). Table 3 also lists the number of items included in each PROM, their domain coverage as per the Wilson and Cleary model, their use of patient input during the initial item generation phase of development, and the number of articles in which the PROM was applied, grouped based on intended application. Seven psychometrically robust PROMs used patient input during the initial item generation phase of development.

**Table 3:** Psychometrically robust PROMs 83 85 87 88 89 90 91 94 95 96 97 98 100 103 104 105

	Number of items	Domains covered as per the Wilson and Cleary model					Patient input	Number of articles in which the PROM was applied, grouped based on intended application			
		*Biological*	*Symptoms*	*Function*	*Health perception*	* Overall quality of life a*	Patient input in initial item generation	To advance knowledge of the condition and/or treatment being investigated	Development of a PROM or psychometric evaluation of an existing PROM	To assess the impact of PROM utilization on patient, process, and/or health service-related outcomes	To investigate determinants influencing PROM implementation, the application of implementation strategies, and subsequent process/outcome evaluations
Generic HRQoL PROMs											
Pediatric Quality-of-Life Inventory 4.0 generic core scales (PedsQL 4.0)	23/21 b						No	30	1	0	0
Dutch-Child-AZL-TNO-Quality-of-Life Questionnaire (DUX-25)	25						No	3	0	0	0
Child Health Questionnaire Child Form 87 (CHQ-CF87)	87						No	4	0	0	0
TNO-AZL Questionnaire for Children's Health-Related Quality of Life (TACQOL)	63						No	1	0	0	0
KIDSCREEN	52/27 c						Yes	2	0	0	0
KINDL-R	40						Yes	1	0	0	0
Condition-specific HRQoL PROMs											
Esophageal-Atresia-Quality-of-Life Questionnaires (EA-QOL)	17/24 d						Yes	3	4	0	0
Hirschsprung's disease Anorectal malformation Quality of Life Questionnaire (HAQL)	39/42 e						Yes	1	0	0	0
Symptom-or domain-specific PROMs											
Strengths and Difficulties Questionnaire (SDQ)	25						No	6	0	0	0
Pediatric Quality-of-Life Inventory Cognitive Functioning Scale (PedsQL CFS)	6						Yes	1	0	0	0
Pediatric Quality-of-Life Inventory Gastrointestinal Symptoms Module (PedsQL GI Module)	58/74 f						Yes	2	0	0	0
Pediatric Perceived Cognitive Functioning Questionnaire (PedsPCF)	43						Yes	1	0	0	0

a If physical, mental, and social dimensions were measured; this was regarded as evaluation of overall quality of life independent of producing a total score or not.

b 23 items for children aged 5–18, 21 for children aged 2–4.

c Long version 52 items, short version 27 items.

d 17 items for children aged 2–7, 24 items for children aged 8–18.

e 39 items for 6, 7 year, and 8–11-year-old children, 41 items for the age group 12–16 years, and 42 items for the age group ≥ 17 years.

f 58 items for the PedsQL Gastrointestinal Symptoms Scale and 74 items for the Symptoms Module.

## Discussion

This narrative review, which had the objective of advancing our knowledge of available PROMs in pediatric surgery, illustrates the ongoing growth in the utilization of PROMs in pediatric surgical research. Our findings, which largely mirror those reported by Besner et al, 11 underscore the gaps in the availability of PROM research for certain pediatric surgical conditions, including anorectal malformations, biliary atresia, congenital diaphragmatic hernia, duodenal atresia, abdominal wall defects, sacrococcygeal teratoma, and short bowel syndrome.

### PROM Development and Suitability for Use

In the article selection and review process, we observed that a notable number of studies utilized self-developed questionnaires, adult PROMs, and in some cases, PROMs originally developed for other conditions. While the use of in-house and other non-psychometrically robust PROMs may indicate good intentions and initial efforts, it is important to note that among the 29 PROMs identified by this narrative review, only 12 can be recommended as measures demonstrating adequate validity and reliability for use in pediatric populations. Among these, only two are condition-specific. The U.S. Food and Drug Administration guidelines published in 2009 13 have had a significant impact on the quality of PROM research in health care and industry-based research. 14 106 107 108 Seven of the 12 psychometrically robust PROMs identified had been developed using patient input in the primary item generation development phase, which is in accordance with current PROM development standards.

There is ongoing debate about whether future development efforts should prioritize condition-specific PROMs. However, considering shared symptomology across different conditions, there may be advantages in utilizing symptom- or domain-specific measurement approaches. 103 In this narrative review, we observed that psychometrically robust symptom- or domain-specific questionnaires were tailored to specific health conditions, encompassing gastrointestinal symptoms, cognitive functioning, and mental health. Notably, a single PROM has been developed to measure HRQoL for two conditions, anorectal malformation and Hirschsprung's disease. 103 However, condition-specific PROMs can be essential for identifying symptom-related challenges which are significant for people living with a particular medical condition. 16 Qualitative interviews and focus groups involving pediatric patients and their parents can be instrumental in enhancing our understanding of these condition-specific experiences. 14 106 107

The evaluation of postoperative colorectal functional outcomes, including aspects like continence, was conducted by the Rintala Bowel Function, 77 Baylor Continence, 78 Cleveland Clinic Fecal Incontinence Severity (or Wexner), 81 82 or Krickenbeck 102 scoring models. These models assess condition-specific outcomes important for patients with anorectal malformations and Hirschsprung's disease. 103 109 110 The Rintala Bowel Function and Krickenbeck postoperative assessment instruments generate clinical scores through the use of questionnaires. The frequent application of these scoring models in research, providing a standardized approach to outcome assessment, is undeniably advantageous. However, these instruments may not meet the criteria for international PROM standards, as a PROM should be completed by the patient “without interpretation by a clinician or someone else.” 13 Additionally, these models lack input from patients and parents during their development. Similarly, the BCS was validated for children with anorectal malformations 78 but was not developed with direct patient input. Neither the Rintala Bowel Function and Krickenbeck scoring models nor the BCS seems to have undergone comprehensive evaluations based on established psychometric criteria of feasibility, validity, and reliability. 26

### Translation and Cross-Cultural Validation

In order to maximize opportunities for rare disease research and quality improvement initiatives facilitated through international collaboration, the translation of PROMs into multiple languages is needed. 111 Generic HRQoL instruments such as the KIDSCREEN, 95 PedsQL 4.0, 83 and KINDL-R 100 are translated in multiple languages. However, the availability of translated condition-specific and symptom- or domain-specific questionnaires varies widely. Previous research has pointed out the necessity to reduce heterogeneity in outcome assessment and promote standardization of PROMs, such as for anorectal malformations, 112 biliary atresia, 7 Hirschsprung's disease, 112 and esophageal atresia, 113 by translating and psychometrically evaluating existing PROMs and developing new PROMs as needed. Just recently, a study reporting on the translation and cultural adaptation of the EA-QOL questionnaire for use in 14 countries was published. 114 Such initiatives can be used as a model for other PROMs in pediatric surgery to standardize outcome assessments cross-culturally. Sets of internationally agreed measures may be of great value in achieving standardized outcome measurement. However, measures included should demonstrate psychometric robustness and meet international PROM standards.

### Impact Assessment and Implementation

Most of the identified PROMs were not applied in studies with a randomized trial design, perhaps due to conceptual and methodological challenges related to the use of PROMs in clinical trials with low-prevalence, heterogeneous conditions. 115 Studies with rigorous study designs, including randomized controlled trial (RCTs), have been advocated for the comprehensive assessment of the impact of PROM use in pediatric clinical care on various critical outcomes. 20 21 The measurement of patient-reported outcomes can aid in assessing the effectiveness of a treatment or the longitudinal monitoring of health status, 116 thereby supporting clinical decision-making. By assessing the relationship between PROMs and other clinical outcome measures, two studies confirmed the complementary value of PROMs for clinical decision-making. However, none of the PROMs were subjected to an evaluation of their impact on patient, process, and/or health service-related outcomes. This observation aligns with findings of previous studies 20 21 reporting on more common pediatric health conditions such as diabetes, asthma, idiopathic arthritis, and cancer. None of the identified PROMs were applied in an implementation study. This identifies impact assessment and implementation as key areas for future focus in the field of PROM research in pediatric surgery.

The field of implementation science offers a structured way of supporting the adoption and evaluation of PROM use in clinical practice. 117 118 Implementation science entails the examination of methods used to facilitate the adoption of evidence-based practices. It draws on theoretical approaches to describe implementation processes, understand and explain factors influencing implementation, and assess the success and effectiveness of implementation strategies. 119

Until recently, it was little known how to effectively implement PROMs in pediatric settings. Scott et al, 22 however, present a list of effective strategies derived from a recent systematic review, based on recognized barriers and facilitators (“determinants”). Barriers included a lack of evidence justifying the psychometric properties of a PROM and a lack of cross-cultural validity and availability of translated versions. There may also be context-specific determinants at play, which can inform the development of context-specific implementation strategies.

The absence of implementation studies, as found in our review and by Besner et al, 11 aligns with Scott et al, 22 who also report no identified implementation studies in the field of pediatric surgery. Application of implementation science principles has gained recognition in pediatric surgery, offering added value. 120 The use of valid and reliable HRQoL questionnaires in the follow-up care of patients with esophageal atresia has been recommended by the European Reference Network for rare Inherited and Congenital Anomalies (ERNICA) and the International Network for Esophageal Atresia (INoEA). 121 122 However, it is important to acknowledge that many HRQoL measures were not primarily designed for individual clinical decision-making, so PROMs should complement clinical data. 15

Comprehensive evaluations of PROM usage in clinical practice, with or without implementation strategies, help determine whether observed changes result from PROM utilization or other factors, such as patient–provider discussions. To enhance understanding, exploring the mechanisms behind outcomes is crucial. For example, increased office visits and endoscopies post-PROM usage may indicate heightened disease awareness or greater willingness to seek care. 123 Health care professionals' understanding of PROM content is also vital for accurate interpretation of changes. 15

### Considerations, Strengths, and Limitations of the Study

While our review adhered to predefined quality criteria, it is not a systematic review and lacks full comprehensiveness. A strength of this review is its broad focus, allowing for the identification of studies pertinent to all stages of the PROM development and application process. In addition to validity, reliability was also considered in determining a PROM's psychometric robustness and the use of patient input in the primary item generation phase was reviewed. Our review does not attempt to provide a full overview of available PROMs recommended for use in pediatric surgery. Rather, it offers new perspectives on PROM research for pediatric surgical conditions, by incorporating lessons from the field of psychometrics. Detailed psychometric evaluations were, however, beyond the article's scope. For example, a PROM's ability to detect change over time (its “responsiveness”) was not assessed. Like Besner et al, 11 we applied the Wilson and Cleary model 27 to classify the PROM content. However, there is potential domain overlap. The review focused solely on children, leaving room for further research on the use of PROMs in adult patients and on outcomes that matter to parents.

## Conclusions and Recommendations to Advance the Field of PROM Research and Promote Successful and Effective Use

While this review demonstrates an increased utilization of PROMs in recent years in pediatric surgical research, this increase seems to be predominantly confined to specific conditions, particularly esophageal atresia and Hirschsprung's disease. Our review underscores the need to broaden the scope of PROM research to include anorectal malformations, biliary atresia, congenital diaphragmatic hernia, duodenal atresia, abdominal wall defects, sacrococcygeal teratoma, and short bowel syndrome.

Our review identified PROMs that are both psychometrically robust and derived from initial pediatric patient input. In future research, valid and reliable PROMs for children and adolescents should be used, and not those developed for adults. Furthermore, their recommended use in research and clinical practice and the need for future PROM development studies will depend on the patient population under investigation and the maturity of the field. This includes the “maturity” of the PROM itself, which can be determined by using the ideal process for PROM development and standardized use described in Supplementary Material S2 .

The need for new instruments and/or the suitability of those existing should be evaluated, considering factors such as condition-specific morbidity and the views and experiences of patients. For some conditions, such as abdominal wall defects, a systematic review of HRQoL in affected children is lacking and therefore warranted. Existing condition-specific PROMs, such as the HAQL and EA-QOL, are recommended for use, translation, and cultural adaptation to standardize outcome assessment and increase the generalizability of study findings. However, a psychometrically robust PROM developed with patient input is warranted to assess colorectal function in rare and complex malformations. For all conditions investigated in this study, the longitudinal assessment of HRQoL is required.

Furthermore, patients with several pediatric surgical conditions could benefit from using existing symptom-specific PROMs, such as the PedsQL GI Module, following appropriate translation and cross-cultural psychometric evaluation. As symptom- or condition-specific PROMs are likely to demonstrate increased sensitivity to disease severity, the PedsQL GI Module, EA-QOL, and HAQL, among others, could be used to incorporate the patient perspective into clinical treatment evaluations.

Altogether, to ensure high-quality data collection from patients, our findings emphasize the importance of applying PROMs appropriately and developing psychometrically robust instruments that are translated and cross-culturally validated. However, we also recommend a future review to provide comprehensive guidance to researchers and clinicians on how to select and use PROMs appropriately for the pediatric surgical conditions investigated.

To explore PROM impact on patient, process and health service-related outcomes, rigorous study designs, such as RCTs, are recommended. Evaluating PROM use can reveal whether changes result from the PROM itself or other factors. The successful and effective implementation of PROMs is also highlighted as a key future research topic in pediatric surgery. Multicenter and international collaboration is vital for PROM development, psychometric evaluation, and implementation. This is also key to fostering research activity in all parts of the world.
