# Supplementary material for: A Narrative Review of Patient-Reported Outcome Measures and Their Application in Recent Pediatric Surgical Research: Advancing Knowledge and Offering New Perspectives to the Field
Source: Eur J Pediatr Surg. 2024 Jan 25;34(2):143–61. doi: 10.1055/s-0043-1778108 (PMC10920019; doi:10.1055/s-0043-1778108)
Supplement: Supplementary file 1 — Supplementary Material [file 10-1055-s-0043-1778108-s2023106784rev.pdf]

Supplementary Material S1

Search strings:

Database searched	Platform	Years of coverage	Records	Records after duplicates removed
Medline ALL	Ovid	1946–present	294	294
Embase	Embase.com	1971–present	451	196
Web of Science Core Collection[a]	Web of Knowledge	1975–present	334	129
Cochrane Central Register of Controlled Trials[b]	Wiley	1992–present	27	10
Total			1,106	629

Note: Science Citation Index Expanded (1975–present); Social Sciences Citation Index (1975–present); Arts & Humanities Citation Index (1975–present); Conference Proceedings Citation Index-Science (1990–present); Conference Proceedings Citation Index-Social Science & Humanities (1990–present); Emerging Sources Citation Index (2005–present). No other database limits were used than those specified in the search strategies.
[a]Exact search turned on in Web of Science Core Collection
[b]Manually deleted abstracts from trial registries.

Medline 294

(Patient Reported Outcome Measures/ OR Quality of Life/ OR Self Report/ OR (((patient*-report* OR self*-report* OR child*-report*) ADJ3 (outcome* OR measure* OR quality-of-life OR symptom* OR experience* OR wellbeing* OR well-being*)) OR prom OR proms OR (quality ADJ3 life) OR hrql OR hrqol OR qol).ab,ti,kw. OR (patient*-report* OR self*-report* OR child*-report* OR parent*-report*).ti.) AND (exp Child / OR exp Infant/ OR Adolescent / OR Pediatrics / OR (child* OR infan* OR adolescen* OR pediatric* OR paediatric*).ab,ti,kw.) AND (Hernia, Abdominal / OR Gastroschisis / OR Hernia, Umbilical / OR Biliary Atresia / OR Hernia, Diaphragmatic / OR Anorectal Malformations / OR Esophageal Atresia / OR Tracheoesophageal Fistula / OR Hirschsprung Disease / OR Short Bowel Syndrome / OR ((abdominal-wall* ADJ3 defect*) OR Gastroschis* OR Omphalocele* OR ((bile-duct* OR Biliary) ADJ3 atresia*) OR (diaphragm* ADJ3 hernia*) OR ((anorectal* OR colon* OR colorect* OR foregut* OR hindgut*) ADJ3 (malformation* OR anomal*)) OR ((duoden* OR esophag* OR oesophag*) ADJ3 atresia*) OR (tracheoesophag* ADJ3 fistula*) OR Hirschsprung* OR (sacrococc* ADJ3 teratoma*) OR short-bowel* OR short-intest*).ab,ti, kw.) AND english.la. AND 2018:2023.(sa_year).

Embase 451

('patient-reported outcome'/de OR 'quality of life'/de OR 'quality of life assessment'/exp OR 'self report'/de OR (((patient*-report* OR self*-report* OR child*-report*) NEAR/3 (outcome* OR measure* OR quality-of-life OR symptom* OR experience* OR wellbeing* OR well-being*)) OR prom OR proms OR (quality NEAR/3 life) OR hrql OR hrqol OR qol):ab,ti,kw OR (patient*-report* OR self*-report* OR child*-report* OR parent*-report*):ti) AND (child/exp OR childhood/exp OR adolescent/de OR adolescence/de OR pediatrics/de OR 'pediatric patient'/de OR (child* OR infan* OR adolescen* OR pediatric* OR paediatric*):ab, ti,kw) AND ('abdominal wall defect'/de OR Gastroschisis/de OR Omphalocele/de OR 'bile duct atresia'/de OR 'diaphragm hernia'/de OR 'congenital diaphragm hernia'/de OR 'anorectal malformation'/de OR 'colon malformation'/de OR 'duodenum atresia'/de OR 'esophagus atresia'/de OR 'tracheoesophageal fistula'/de OR 'Hirschsprung disease'/de OR 'sacrococcyx teratoma'/de OR 'short bowel syndrome'/de OR ((abdominal-wall* NEAR/3 defect*) OR Gastroschis* OR Omphalocele* OR ((bile-duct* OR Biliary) NEAR/3 atresia*) OR (diaphragm* NEAR/3 hernia*) OR ((anorectal* OR colon* OR colorect* OR foregut* OR hindgut*) NEAR/3 (malformation* OR anomal*)) OR ((duoden* OR esophag* OR oesophag*) NEAR/3 atresia*) OR (tracheoesophag* NEAR/3 fistula*) OR Hirschsprung* OR (sacrococc* NEAR/3 teratoma*) OR short-bowel* OR short-intest*):ab,ti,kw) NOT [conference abstract]/lim AND [english]/lim AND [2018-2023]/py

Web of Science 334

TS = (((((patient*-report* OR self*-report* OR child*-report*) NEAR/2 (outcome* OR measure* OR quality-of-life OR symptom* OR experience* OR wellbeing* OR well-being*)) OR prom OR proms OR (quality NEAR/2 life) OR hrql OR hrqol OR qol) OR (patient*-report* OR self*-report* OR child*-report* OR parent*-report*):ti) AND ((child* OR infan* OR adolescen* OR pediatric* OR paediatric*)) AND (((abdominal-wall* NEAR/2 defect*) OR Gastroschis* OR Omphalocele* OR ((bile-duct* OR Biliary) NEAR/2 atresia*) OR (diaphragm* NEAR/2 hernia*) OR ((anorectal* OR colon* OR colorect* OR foregut* OR hindgut*) NEAR/2 (malformation* OR anomal*)) OR ((duoden* OR esophag* OR oesophag*) NEAR/2 atresia*) OR (tracheoesophag* NEAR/2 fistula*) OR Hirschsprung* OR (sacrococc* NEAR/2 teratoma*) OR short-bowel* OR short-intest*))) AND DT = (article) AND PY = (2018-2023)

 © 2024. The Author(s).

Cochrane 27

((((patient* NEXT report* OR self* NEXT report* OR child* NEXT report*) NEAR/3 (outcome* OR measure* OR quality-of-life OR symptom* OR experience* OR wellbeing* OR well-being*)) OR prom OR proms OR (quality NEAR/3 life) OR hrql OR hrqol OR qol):ab,ti,kw OR (patient* NEXT report* OR self* NEXT report* OR child* NEXT report* OR parent* NEXT report*):ti) AND ((child* OR infan* OR adolescen* OR pediatric* OR paediatric*):ab,ti,kw) AND (((abdominal-wall* NEAR/3 defect*) OR Gastroschis* OR Omphalocele* OR ((bile-duct* OR Biliary) NEAR/3 atresia*) OR (diaphragm* NEAR/3 hernia*) OR ((anorectal* OR colon* OR colorect* OR foregut* OR hindgut*) NEAR/3 (malformation* OR anomal*)) OR ((duoden* OR esophag* OR oesophag*) NEAR/3 atresia*) OR (tracheoesophag* NEAR/3 fistula*) OR Hirschsprung* OR (sacrococc* NEAR/3 teratoma*) OR short-bowel* OR short-intest*):ab,ti,kw)

Supplementary Material S2 Ideal process for PROM development and standardized use[a]

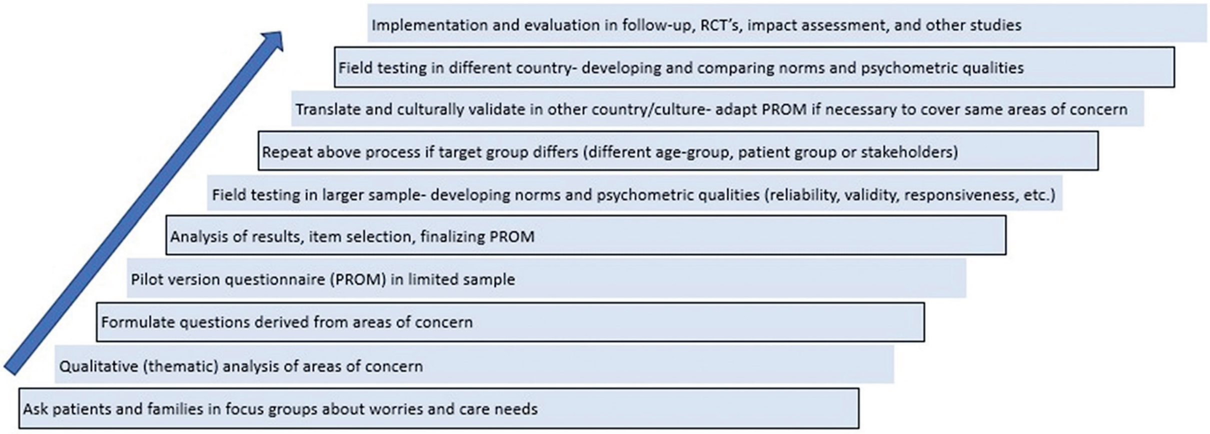

[a]Multicenter, international collaboration is important throughout.

 © 2024. The Author(s).
